# Three-Dimensional Bioprinting of Tarsal Plates with Adipose-Derived Mesenchymal Stem Cells: Evaluation of Meibomian Gland Reconstruction in a Rat Model

**DOI:** 10.3390/biomedicines12112567

**Published:** 2024-11-09

**Authors:** Hyunkyu Lee, Yoon Hee Park, Hyo Jin Kang, Hwa Lee

**Affiliations:** 1 Department of Ophthalmology, Korea University College of Medicine, Anam Hospital, Seoul 02841, Republic of Korea; leehg313@gmail.com; 2 Medical Science Research Center, Korea University College of Medicine, Ansan Hospital, Ansan 15355, Republic of Korea; yunisto@korea.ac.kr; 3 Department of Biomedical Laboratory Science, Honam University, Gwangju 62399, Republic of Korea; 4 Department of Ophthalmology, Korea University College of Medicine, Ansan Hospital, Ansan 15355, Republic of Korea

**Keywords:** tarsal plate, 3D bioprinting, tissue engineering, adipose-derived mesenchymal stem cells, meibocyte, meibomian gland

## Abstract

**Background:** The aim of this study was to develop 3D-bioprinted scaffolds embedded with human adipose-derived stem cells (hADSCs) to reconstruct the tarsal plate in a rat model. **Methods:** Scaffolds were printed using a 3D bioprinter with a bioink composed of atelocollagen and alginate. hADSCs (5 × 10^5^ cells/mL) were embedded within the bioink. A total of 30 male Sprague Dawley (SD) rats (300 g) were divided into three groups: group 1 (normal control, *n* = 10), group 2 (3D-bioprinted scaffolds, *n* = 10), and group 3 (3D-bioprinted scaffolds with hADSCs, *n* = 10). Four weeks after surgery, a histopathological analysis was performed using hematoxylin and eosin (H&E) staining, Masson’s trichrome (MT) staining, and immunofluorescence staining. Gene expression of SREBP-1, PPAR-γ, FADS-2, and FAS was assessed via real-time polymerase chain reaction (PCR). **Results:** No abnormalities were observed in the operated eyelids of any of the 30 rats. The histopathological analysis revealed lipid-secreting cells resembling meibocytes in both group 2 and group 3, with more pronounced meibocyte-like cells in group 3. Immunofluorescence staining for phalloidin expression showed a significant increase in group 3. Additionally, the RNA expression of SREBP-1, PPAR-γ, FADS-2, and FAS, all related to lipid metabolism, was elevated in group 3. **Conclusions:** The 3D-printed scaffolds combined with hADSCs were effective for tarsal plate reconstruction, with the hADSCs notably contributing to the generation of cells associated with lipid metabolism.

## 1. Introduction

The tarsal plate is the most important component of the eyelid and is composed of dense connective tissue, abundant elastic fibers, and numerous meibomian glands [1,2]. In addition, the tarsal plate is present in both the upper and lower eyelids and not only serves as a structural support for maintaining the shape of the eyelid but also contributes to the stabilization of the tear film and prevents the cornea from drying out by forming the lipid layer of the tear layer, with lipid components secreted from the meibomian gland in the tarsal plate [3,4].

There are various clinical problems associated with the tarsal plate, which plays an important role. Various clinical issues that can occur in the tarsal plate include invasion by malignant eyelid tumors such as basal cell carcinoma, squamous cell carcinoma, sebaceous gland carcinoma, and malignant melanoma, direct damage due to eyelid or orbital trauma, and functional loss due to severe inflammation of the meibomian glands [5]. In the case of malignant eyelid tumors, reconstruction of the defect is necessary because the entire eyelid or part of the eyelid, including normal tissue, is resected [5]. For eyelid reconstruction, a substitute that can serve as the tarsal plate is essential, and hard palate mucosa and nasal cartilage are used [6,7]. Spacer grafts incorporating acellular dermis have recently been used as autologous, allogenic, or alloplastic materials inserted into the posterior lamella of the eyelid to provide structural support [8,9,10]. However, there are limitations, such as large shrinkage of the graft after surgery, a limited range of tissue, and immune rejection, and problems that adversely affect the stability of the tear film because the secretion function of the meibomian gland cannot be replaced [6,7]. Therefore, it is crucial to develop a tarsal plate replacement with specific lipid secretion activity.

A method for producing customized three-dimensional (3D) objects using computer-aided design is 3D bioprinting [11]. This innovative technology encompasses various methods, each suited to different types of tissues and applications. In this study, we specifically utilized extrusion-based 3D bioprinting to create scaffolds for tissue engineering, as it allows for greater flexibility in the material selection and control over the scaffold’s porosity and structure, which cannot easily be achieved with conventional techniques [12,13]. Bioprinting and bio-scaffolding of biological structures with intricate architectures can leverage the advantageous properties of additive manufacturing technology, enabling the creation of complex and precise scaffolds [14,15]. These benefits make 3D bioprinting an excellent choice for creating fragile and complicated tissues, such as the cornea [16,17].

The complexity of the tarsal plate tissue and the absence of acceptable alternatives currently impose limitations on tarsal plate tissue engineering, a crucial aspect of eyelid reconstruction. An ideal tarsal plate substitute must possess the strength, flexibility, and surface qualities found in natural tarsal tissue. Moreover, it should be easy to obtain and handle and compatible with other tissue [18]. Tissue reconstruction using 3D bioprinting offers a solution to these limitations, making it possible to create a simple but highly suitable framework for bioprinting and bio-scaffolding biological objects through additive manufacturing technology [18]. Although various studies have explored the application of these technologies in the ophthalmic field, the specific types of cells that can be cultured within this framework are diverse and have not yet been established fully. Furthermore, while structural support plays a crucial role in maintaining the eyelid’s shape, the secretion of lipid components from the meibomian glands is equally significant. Despite these factors, there has been no successful example of tarsal plate transplantation using 3D bioprinting applied to humans.

In this study, human adipose-derived mesenchymal stem cells (hADSCs) were incorporated into 3D-bioprinted scaffolds with the aim of reconstructing the tarsal plate and promoting glandular function, particularly lipid secretion from the meibomian glands. We hypothesized that hADSCs would be suitable for this application due to their ability to promote tissue regeneration, their multipotent nature, and their immunomodulatory properties, which make them promising candidates for tissue engineering. This hypothesis was made because, to date, there have been few attempts at tarsal plate reconstruction using 3D bioprinting. Our approach was designed to address both the structural and functional needs provided by the tarsal plate. The therapeutic potential of these bioprinted scaffolds was evaluated in a rat model with eyelid removal, where scaffold engraftment and lipid secretion function were assessed. The results confirmed the biocompatibility and potential efficacy of the bioprinted scaffolds, supporting their possible use in tarsal plate tissue engineering.

## 2. Materials and Methods

### 2.1. Preparation of the 3D-Bioprinted Scaffolds for the Tarsal Plate

hADSCs were obtained from ScienCell Research Laboratories (Carlsbad, CA, USA). The cells were cultured in Mesenchymal Stem Cell Medium (ScienCell Research Laboratories, Carlsbad, CA, USA). The hADSCs were maintained in a humidified incubator at 37 °C with 5% CO_2_. The medium was changed every 2–3 days, and the cells were passaged at approximately 80–90% confluency using 0.25% trypsin–EDTA. All the experiments were performed using cells between passages 3 and 6 to ensure consistent behavior.

**Figure 1:**
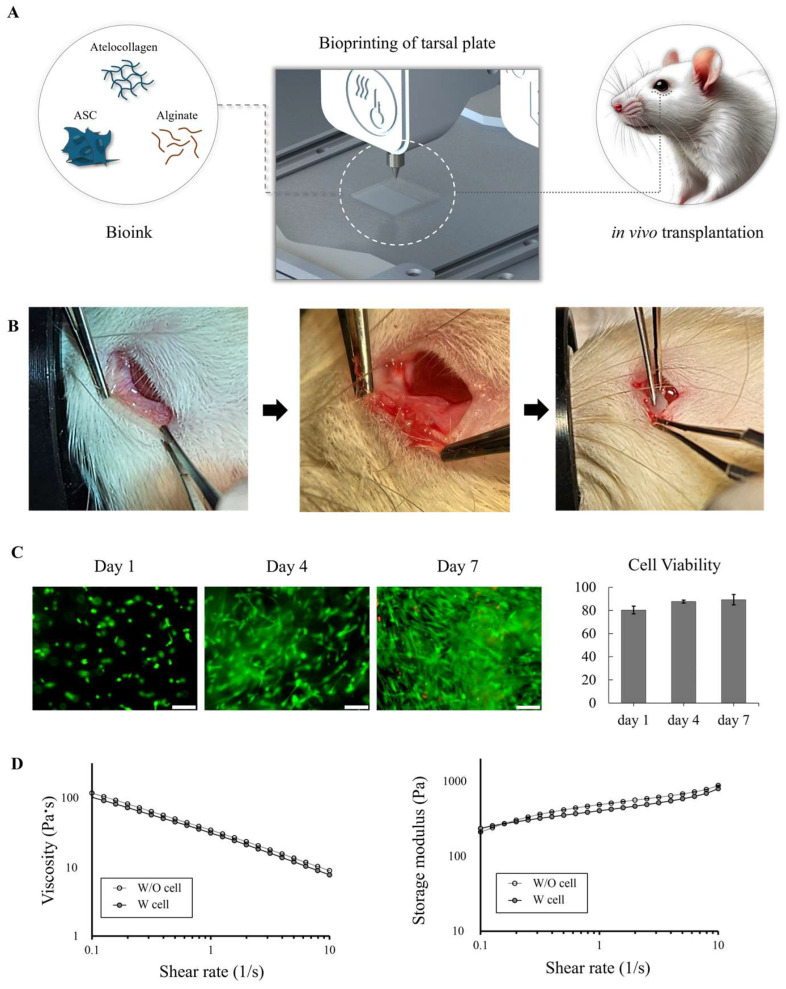
Three-dimensional bioprinting of tarsal plate for in vivo transplantation. (**A**) Schematic of bioink preparation using atelocollagen, alginate, and adipose-derived stem cells (ASCs), followed by the bioprinting of the tarsal plate structure and its in vivo transplantation into a rat model. (**B**) Stepwise procedure for the in vivo transplantation of the bioprinted tarsal plate into the rat model. (**C**) Bioprinted ASCs were observed under fluorescence microscopy over 7 days; cell viability was assessed by staining live cells (green) and dead cells (red). (**D**) Rheological properties of bioinks with and without cells. The storage modulus (Pa) at 1 Hz was 376.34 ± 19.95 Pa with cells and 428.67 ± 30.48 Pa without cells.

The scaffolds were printed using a 3D extrusion-based bioprinter (Baobab Root-1, Baobab Healthcare Inc., Ansan, Republic of Korea) (Figure 1A). This printer allows for a resolution of approximately 1 µm, with a maximum printing speed of 3000 cm/min and accuracy within ±1 µm, which is crucial for maintaining the detailed structure of the scaffolds. Extrusion-based bioprinting was selected due to its flexibility in accommodating a wide range of bioinks with varying viscosities, offering greater diversity in the material selection. The bioink was prepared by combining 3 wt/v% neutralized atelocollagen (4 °C, Baobab Healthcare Inc., Republic of Korea) and 3 wt/v% alginate (viscosity > 2000 cP, 4 °C, Sigma-Aldrich, St. Louis, MO, USA) at a ratio of 4:1 (Figure 1A). The hADSCs (5 × 10^5^ cells/mL) were embedded within the bioink (Figure 1A). The bioink was then printed through a tapered nozzle (22G) at 45 kPa on a 37 °C printing base with a crosshead speed of 200 mm/min. The printed scaffolds had dimensions of 25 mm in length, 25 mm in width, and 0.4 mm in thickness. To cross-link the alginate, the printed samples were soaked in 200 mM CaCl_2_ for 2 min, followed by washing them three times with phosphate-buffered saline (PBS) and incubating them at 37 °C for 1 h to gelate the atelocollagen.

### 2.2. Cell Viability

The cell viability within the bioprinted structures was assessed using a live/dead viability assay (Thermo Fisher Scientific, Eugene, OR, USA). Bioprinted constructs were incubated with a solution containing calcein-AM (2 μM, to stain live cells) and ethidium homodimer-1 (4 μM, to sta It was written in MS Word using Formula format, which were in italics. The italics have been removedin dead cells) for 30 min at 37 °C. Fluorescence microscopy images were taken on days 1, 4, and 7 post-printing. Green fluorescence indicated live cells, while red fluorescence indicated dead cells. Random images were taken from 5 different regions of each sample for analysis. The percentage of viable cells was calculated from these images using ImageJ 1.54g software. Cell viability was determined using the following formula:

$$Cell\;Viability\left(\%\right)=\left(\frac{Number\;of\;live\;cells}{Total\;number\;of\;cells\left(live+dead\right)}\right)\times 100$$

Randomly captured images from five different regions were used to assess the overall cell viability, ensuring representative data from various parts of the bioprinted structure.

### 2.3. Rheological Characterization

The rheological properties of the bioinks, with and without cells, were measured using a rotational rheometer (Anton Paar, MCR 102e, Graz, Austria). The viscosity and storage modulus were determined at 4 °C over a shear rate range of 0.1 to 10 s^−1^. Viscosity measurements were made to assess the shear-thinning behavior of the bioinks, while the storage modulus values were used to evaluate the stiffness of the bioinks.

### 2.4. The Animals and Modeling of the Removal of the Posterior Lamella 

Sprague Dawley male rats (SD, 8 weeks old, 280–300 g) were purchased from ORIENT BIO (Seongnam, Republic of Korea) and maintained according to the Association for Assessment and Accreditation of Laboratory Animal Care International system. All animal experiments conformed to the International Guide for the Care and Use of Laboratory Animals and were approved by the Institutional Animal Care and Use Committee of the Korea University College of Medicine (IACUC No. KOREA-2021-0209). A total of 30 male Sprague Dawley (SD) rats (300 g, OrientBio, Seong-nam, Republic of Korea) were randomly divided into three groups: normal control (group 1, *n* = 10), 3D-bioprinted scaffolds (group 2, *n* = 10), and 3D-bioprinted scaffolds combined with ADSCs (group 3, *n* = 10).

Inhalation anesthesia was administered using a 1 L/min gas mixture with 90% medical oxygen and 2% isoflurane to the rats in groups 2 and 3, from whom the posterior lamellae were removed and into whom the tarsal plate implants were implanted. Before surgery, an aseptic 3D-bioprinted scaffold was prepared for implantation into the tarsal plate defect (3 mm in length, 2 mm in width, and 0.4 mm in thickness). After everting the lower eyelids of the anesthetized rats to expose the palpebral conjunctiva, an incision was made at the boundary between the anterior lamella and the posterior lamella of the lower eyelids of the anesthetized rats, as observed under a microscope (Figure 1A,B). Subsequently, the anterior and posterior lamellae, along with the tarsal plate, were separated using blunt dissection (Figure 1B). Subsequently, an incision was made to separate the inferior eyelid tarsal plate border from the conjunctiva and the lower eyelid retractor muscles (Figure 1B). An eyelid tarsal defect plate 3 mm in length, 2 mm in width, and 0.4 mm in thickness was then made with microscissors (Figure 1B). In group 2, the posterior lamella was removed, and a 3D-bioprinted scaffold was implanted, whereas in group 3, a 3D-bioprinted scaffold based on hADSCs was implanted (Figure 1B). Antibiotic eye drops were applied four times daily, and antibiotic eye ointment was administered twice daily for four weeks following 3D-bioprinted scaffold implantation to prevent infection. Additionally, the engraftment state of the implanted eyelids was evaluated daily. The rats from all the groups were sacrificed four weeks after the 3D-bioprinted scaffold transplantation to obtain their eyelid tissue.

### 2.5. Histopathological Assessment

The tissues were fixed in 10% formalin and embedded in paraffin. The tissues were routinely processed and cut into 4–5 μm thick sections. The sections were deparaffinized in xylene at room temperature and stained with hematoxylin and eosin (Cancer Diagnostics Inc., Durham, NC, USA) according to the manufacturer’s instructions. Masson’s trichrome staining (BBC Biochemical, Mount Vernon, WA, USA) was performed following the manufacturer’s protocol. Briefly, the deparaffinized sections were fixed in Bouin’s solution for 1 h at 56 °C and stained with ClearView Iron Hematoxylin working solution for 10 min. Subsequently, the tissues were stained with Biebrich scarlet–acid fuchsin solution (2 min), phosphomolybdic–phosphotungstic acid solution (10 min), aniline blue solution (3 min), and 1% acetic acid solution (2 min). The ECM, collagen, and other connective tissue elements were stained blue, and smooth muscle was stained red. The tissue sections were imaged using PANNORAMIC™ Digital Slide Scanners (3DHISTECH Ltd., Budapest, Hungary).

### 2.6. Phalloidin Staining for Filamentous Actin

Phalloidin staining was performed to visualize the distribution and organization of actin filaments by specifically binding to filamentous actin (F-actin). Tissue sections were deparaffinized in xylene and rehydrated in a graded ethanol series. After heat-induced epitope retrieval in Tris-EDTA HIER Solution, at a pH of 9.0 (Scytek Laboratories, Inc., Logan, UT, USA), for 40 min, the sections were incubated with 3% bovine serum albumin blocking reagent for 10 min at room temperature. After blocking, the sections were incubated with Alexa Fluor 488™ phalloidin (Life Technologies, Eugene, OR, USA) for 2 days at 4 °C. The sections were mounted with DAPI-containing mounting medium (Vector Laboratories Inc., Burlingame, CA, USA) and observed under an inverted microscope (Zeiss MicroImaging GmbH, Jena, Germany). The F-actin expression was quantified using ImageJ software, and the stained area was measured as a percentage and then compared among the groups.

### 2.7. RNA Extraction and Real-Time PCR

The total RNA extraction from the paraffin-embedded (FFPE) samples was performed using the RNeasy FFPE Kit (Qiagen, Hilden, Germany) according to manufacturer’s instructions. Briefly, the paraffin blocks were cut to a 5 μm thickness, and the paraffin was removed using a deparaffinization solution (Qiagen, Hilden, Germany). For protein hydrolysis, the tissue was lysed in Buffer PKD and Proteinase K at 56 °C for 15 min, followed by heat treatment at 80 °C for 15 min to reverse the formaldehyde cross-linking. And then, the RNA was purified using RNeasy MinElute spin columns. The RNA was eluted in RNase-free water, and its concentration was assessed using a NanoDrop spectrophotometer (Thermo Fisher Scientific, Waltham, MA, USA). The RNA was reverse-transcribed using amfiRivert cDNA Synthesis Platinum Master Mix (GenDEPOT, Barker, TX, USA). Reverse transcription was performed in a thermocycler (Eppendorf, Hamburg, Germany) at 65 °C for 1 min, 25 °C for 50 min, 55 °C for 60 min, and 85 °C for 1 min.

The primer sequences for real-time PCR are shown in Table 1. The real-time PCR reactions were carried out in triplicate to a final volume of 25 μL in 2× SYBR^®^ Green PCR MasterMix (Applied Biosystems, Foster City, CA, USA). Amplification was performed using an Applied Biosystems Cycler (Foster City, CA, USA).

### 2.8. Statistical Analysis

Quantitative data are given as means ± standard deviation. Differences between groups were evaluated using a one-way analysis of variance, followed by Dunn’s multiple comparison post hoc tests. *p*-values < 0.05 were considered indicative of statistical significance. The statistical analysis was performed using Prism v.5.01 (GraphPad Software, Inc., La Jolla, CA, USA) and Statistical Package for Social Science software, version 22 (SPSS, Chicago, IL, USA).

## 3. Results

### 3.1. Three-Dimensional Bioprinting, Cell Viability, and Rheological Properties

Three-dimensional bioprinting of the tarsal plate structure was successfully achieved using the formulated bioink, as illustrated in Figure 1, which shows the smooth bioprinting process with consistent bioink extrusion and no nozzle clogging or deformation of the printed structure. The printed tarsal plate maintained its predefined dimensions and structural integrity during both the printing process and subsequent incubation.

The cell viability within the 3D-bioprinted scaffold was assessed over a 7-day period using a live/dead fluorescence assay. High cell viability was observed immediately post-printing, with over 90% of the cells alive, as indicated by green staining. By day 4, cell proliferation was evident, and by day 7, the cell density had further increased, with the cells remaining uniformly distributed. Quantitative analysis from five randomly selected regions confirmed consistent cell viability above 90% across the entire construct throughout the 7 days (Figure 1C).

Rheological analysis of the bioink demonstrated its shear-thinning behavior, with its viscosity decreasing as the shear rate increased. Both the cell-laden and cell-free bioinks exhibited similar viscosity profiles, indicating that the presence of cells had little impact on its flow properties. The storage modulus, which reflects elasticity, also showed no significant differences between the bioinks with and without cells. These findings suggest that the inclusion of the hADSCs did not compromise the rheological properties of the bioink, which maintained both its printability and mechanical stability (Figure 1D).

### 3.2. The State of Eyelid Implantation

**Figure 2:**
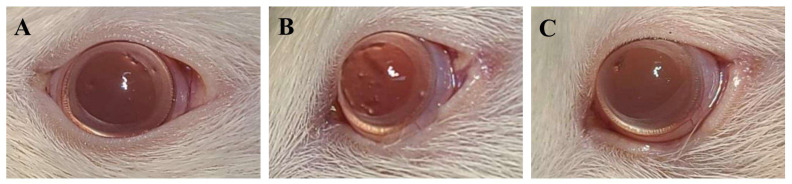
Comparison of the state of the operated eyelids and corneas among the three groups. (**A**) Normal control group, (**B**) 3D-bioprinted scaffold group, and (**C**) 3D-bioprinted scaffold with hADSC group.

There were no intraoperative complications in any of the 30 rats, and follow-up observation for four weeks before sacrifice revealed no abnormalities in their general condition or the operated eyelids and corneas (Figure 2).

### 3.3. Histological Analyses

**Figure 3:**
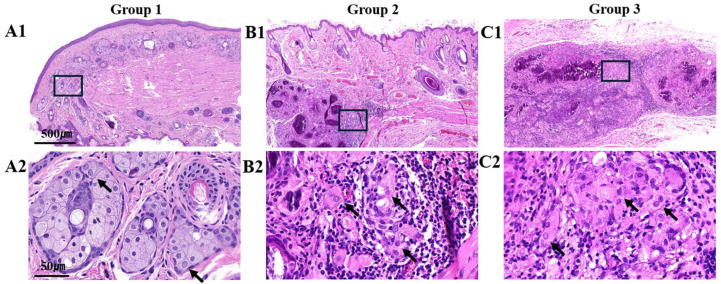
Representative hematoxylin and eosin staining images of the tarsal plate in the posterior lamella. (**A1**) Normal control group: The overall structure of the eyelid, from the anterior to the posterior lamella, is clearly visible. (**A2**) Numerous normal meibomian glands (arrows) are observed within the tarsal plate of the posterior lamella (black square). (**B1**) Three-dimensional-bioprinted scaffold group: The scaffold implanted beneath the muscle layer of the anterior lamella is visible. (**B2**) Cells resembling mucus-secreting cells (arrows) are present within the scaffold (black square). (**C1**) Three-dimensional-bioprinted scaffold with hADSC group: The scaffold implanted beneath the muscle layer of the anterior lamella is visible. (**C2**) Glandular epithelial cells with abundant cytoplasm, morphologically similar to meibomian gland epithelial cells, are observed (black square, arrows). Tissue sections were imaged using digital slide scanners.

As a result of the H&E staining performed in the sacrificed rats after 4 weeks of follow-up after surgery, it was observed that the eyelid thickness in groups 2 and 3 was well maintained compared to that in the normal eyelids of group 1 (Figure 3(A1,B1,C1)). H&E staining of the rats in group 1 revealed normal skin structures, glandular cells, and muscle layers (Figure 3(A1)). A significant number of normal meibomian glands were observed in the tarsal plate situated within the posterior lamella (Figure 3(A2)). In addition, differentiating meibocytes emerged from the basement membrane of the meibomian gland (Figure 3(A2)). As meibocytes mature, they become white and acquire the ability to secrete lipids. In group 2, the overall appearance of the scaffold implanted under the muscle layer of the anterior lamella was observed (Figure 3(B1)). Significant lymphocytic infiltration was observed at the site of the scaffold implantation. In addition, within the scaffold, some cells resembling lipid-secreting cells were observed, while the majority exhibited fibroblast-like shapes (Figure 3(B2)). In group 3, similar to in group 2, the scaffold implanted beneath the muscle layer of the anterior lamella was observed (Figure 3(C1)). Glandular epithelial cells with abundant cytoplasm were observed, and their morphology strongly resembled that of the lipid-secreting cells (Figure 3(C2)). Infiltration of inflammatory cells, mainly lymphocytes, was also confirmed in the periphery of the transplanted area (Figure 3(C2)). In summary, neither group 2 nor group 3 showed cells within the scaffold that were similar to normal meibocytes. However, in group 3, compared with group 2, it was easier to observe cells with many lipid components and a morphology resembling lipid-secreting cells.

Group 1 had 177 ± 24.14 cells/10 HPF, group 2 had 26 ± 19.49 cells/10 HPF, and group 3 had 137.52 ± 19.56 cells/10 HPF, which was statistically significant, according to quantitative measurements made using an ×400-magnification slide scanner of cells capable of secreting fat comparable to meibocytes (Table 2). Additionally, using Tukey’s test to assess the statistical significance of the differences in the cell counts across the groups, it was found that there was a significant difference between groups 1 and 2 and between groups 2 and 3. The comparison of groups 1 and 3 showed a statistically significant difference in cell number (*p* = 0.047). 

**Figure 4:**
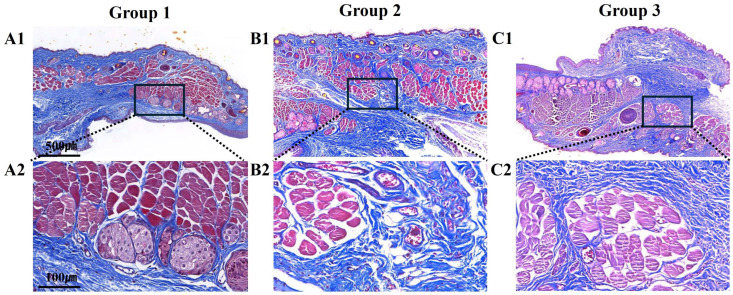
Representative Masson’s trichrome staining images of the tarsal plate in the posterior lamella. Masson’s trichrome staining shows skeletal muscle (red) regeneration and collagen (blue) deposition in all groups. (**A1**) Normal control group (**A2**) An enlarged image of the black square in A1, (**B1**) 3D-bioprinted scaffold group, (**B2**) An enlarged image of the black square in B1, (**C1**) 3D-bioprinted scaffold with hADSC group, and (**C2**) An enlarged image of the black square in (**C1**). Tissue sections were imaged using digital slide scanners.

The results of MT staining were generally the same as those of H&E staining. Several normal meibomian glands were found in the posterior lamellae of group 1 (Figure 4(A1)). Additionally, blue staining was noted diffusely throughout the eyelids (Figure 4(A2)). Fibrosis was observed in group 2 (Figure 4(B1,B2)). In addition, group 2 showed blue staining around the transplant site, indicating tissue remodeling, as collagen and other connective tissues were stained; the density was lower than that in group 1 (Figure 4(B1,B2)). In group 3, tissue remodeling was observed, similar to that in group 2, with connective tissue showing blue staining both within and around the implanted scaffold (Figure 4(C1,C2)). However, in group 3, the collagen density at the graft site was higher than that in group 2, suggesting better tissue remodeling in group 3 (Figure 4(C1,C2)).

### 3.4. F-Actin Expression by Phalloidin Staining

**Figure 5:**
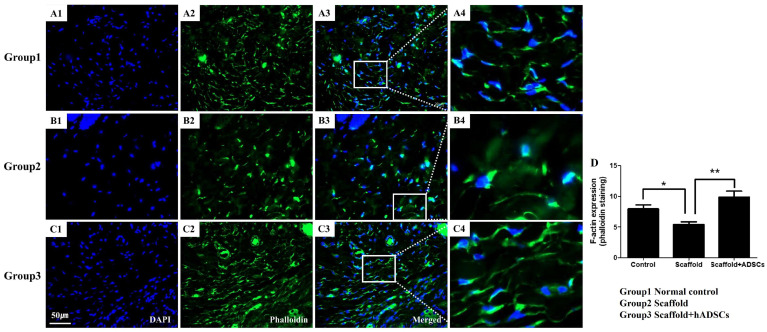
F-actin expression by phalloidin staining. F-actin was stained using fluorescent phalloidin conjugates. (**A**) Normal control group, (**B**) 3D-bioprinted scaffold group, and (**C**) 3D-bioprinted scaffold with hADSC group. The sections were stained with (1) DAPI (blue) and (2) anti-phalloidin (green). (3) Merged and (4) An enlarged image of the white square. (**D**) F-actin expression was quantified using ImageJ software and was measured as a percentage and then compared among the groups (* *p* < 0.05, ** *p* < 0.01; *n* = 6 per group).

To confirm the presence and arrangement of F-actin, a type of actin filament that acts as a cytoskeleton in the posterior lamella, the expression of F-actin (green fluorescence) was confirmed. Expression of F-actin in the normal posterior lamella indicates that F-actin exhibits a stable cytoskeletal structure and normal cell activity (Figure 5A). In the scaffold transplantation group (group 2), F-actin expression was rarely observed, and it was expressed relatively less than in the normal control (group 1) and the scaffold with hADSC group (group 3), and this shows that the tarsal plate was not completely reconstructed (Figure 5B). In the 3D-bioprinted scaffold group with hADSCs, F-actin expression was clearly observed, and it was confirmed to be similar to or even increased compared to that in the normal control (Figure 5C). The percentage of F-actin expression quantified by ImageJ software was significantly increased in the normal control (7.97 ± 0.62% *, * *p* < 0.05) and hADSC + scaffold (9.89 ± 0.95% **, ** *p* < 0.01) groups compared to the scaffold transplantation (5.42 ± 0.41%) group (Figure 5D).

### 3.5. Lipid-Metabolism-Related Genes

**Figure 6:**
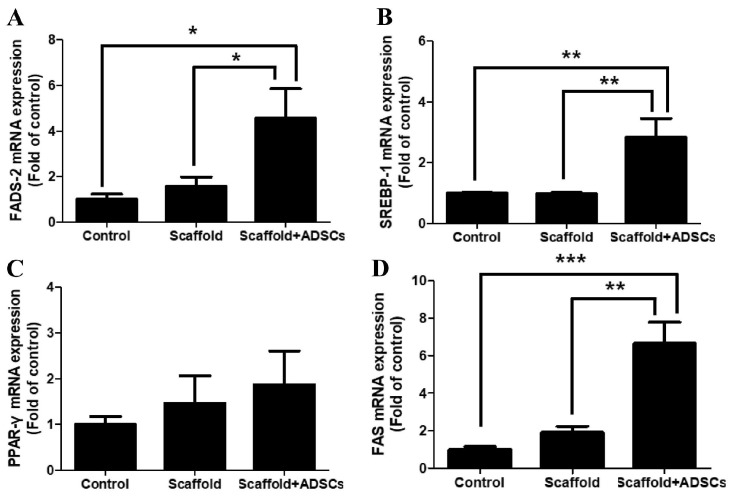
Expression of lipid-metabolism-related genes. (**A**) FADS-2, (**B**) SREBP-1, (**C**) PPAR-γ, and (**D**) FAS were determined by real-time polymerase chain reaction. Data are presented as means ± SEM relative to β-actin mRNA levels (* *p* < 0.05, ** *p* < 0.01, *** *p* < 0.001; *n* = 6 per group). FADS-2; fatty acid desaturase-2, SREBP-1; sterol regulatory element-binding protein-1, PPAR-γ; peroxisome proliferator-activated receptor-γ, FAS; fatty acid synthase.

To determine whether lipid metabolism was present after 3D-bioprinted scaffold transplantation in an animal model of posterior lamella removal, the expression of related genes, such as SREBP-1, PPAR-γ, FADS-2, and FAS, was evaluated using real-time PCR. In the normal control (groups 1) and 3D-bioprinted scaffold (group 2) groups, the expression of SREBP-1, PPAR-γ, FADS-2, and FAS RNA was relatively similar. However, in the 3D-bioprinted scaffold with hADSC group (group 3), the mRNA expression of SREBP-1, FADS-2, and FAS was significantly increased compared to that in groups 1 and 2 (Figure 6A,B,D). The expression of PPAR-γ mRNA was insignificant between the groups and was not statistically significant (Figure 6C).

## 4. Discussion

Due to the eyelid’s complex three-layer structure, reconstruction is critical when it is injured [19]. Choosing the appropriate technique can be difficult, especially for the lower lid, which has both cosmetic and functional significance [19]. Various therapeutic options exist for full-thickness eyelid injuries, but larger defects require more complex surgical methods [19]. Numerous eyelid reconstruction techniques have been developed, typically depending on the defect size [19]. However, unfamiliar procedures may take longer and present limitations, such as the frequent need for additional surgeries [19,20]. Moreover, finding a tarsal plate replacement is challenging due to its dual role in secreting lipids into the tear film and maintaining the eyelid’s structure. Thus, the main goal of tissue engineering is to create an ideal tarsal plate substitute with properties matching the thickness, surface characteristics, strength, and flexibility of the natural tissue.

In this study, we developed a 3D-printed scaffold embedded with hADSCs to evaluate whether lipid-secreting cells or structures were formed within it. To address the challenges in tarsal plate reconstruction, we fabricated a collagen-based 3D-bioprinted scaffold with hADSCs to enhance tissue regeneration and restore lipid metabolism through meibomian gland cell reconstruction. The cell viability in the scaffold exceeded 90% immediately post-printing, and after 7 days, cell proliferation, increased density, and a uniform distribution were observed. Thus, the scaffold demonstrates a reliable bioprinting process with confirmed cell survival and growth stability.

In the animal experiments, a normal control group was used instead of a posterior lamella removal model to assess how well the tarsal plate was reconstructed and whether meibomian-gland-associated cells regenerated after the transplantation of the 3D-bioprinted scaffolds with hADSCs. Histological analysis of the normal tarsal plate revealed typical adipose glands and adipocytes differentiated from the basement membrane. In the scaffold-only group, significant lymphocyte infiltration was observed, and while some lipid-secreting-cell-like structures were present, most of the cells resembled fibroblasts. In contrast, the animals receiving the bioprinted scaffold with hADSCs exhibited glandular epithelial cells with structures closely resembling lipid-secreting cells, indicating that hADSCs contributed significantly to tissue regeneration. The beneficial effects of hADSCs on tissue repair have been well documented [21,22]. hADSCs secrete bioactive molecules, including growth factors, cytokines, and extracellular vesicles, which promote tissue regeneration and reduce inflammation [22,23,24,25]. Thus, transplanting a bioprinted scaffold with hADSCs is more effective for tissue regeneration than using a scaffold alone.

Phalloidin selectively binds to F-actin, a key cytoskeletal filament, enabling the visualization of its distribution through fluorescent staining [26]. In the normal posterior lamella, F-actin helps maintain cellular structure, shape, and mechanical integrity. Phalloidin staining reveals uniformly distributed actin filaments in normal tissues [26]. Collagen-based biomaterials or scaffolds that mimic the extracellular matrix (ECM) can promote F-actin-driven cell migration and ECM remodeling [27]. Fibroblasts or hADSCs that are primed to reorganize collagen and accelerate tissue regeneration through F-actin can improve wound healing. These cells enhance ECM remodeling by dynamically altering the actin cytoskeleton and collagen deposition [28]. In our results, the normal control group (group 1) showed clear F-actin expression (green fluorescence), whereas the scaffold-only group (group 2) exhibited reduced F-actin expression. However, the group that received the bioprinted scaffold with hADSCs (group 3) displayed F-actin expression comparable to that in normal tissue. In group 2, although the scaffold successfully integrated, F-actin migration and ECM remodeling were not yet fully completed. In contrast, group 3 demonstrated more F-action and a stable cytoskeletal structure, indicating more effective tissue remodeling

To assess lipid metabolism alongside tissue regeneration after the transplantation of the bioprinted scaffolds or the bioprinted scaffolds with hADSCs, the expression of lipid-metabolism-related genes (SREBP-1, PPAR-γ, FADS-2, and FAS) was examined. FADS2 is involved in fatty acid desaturation, converting saturated into unsaturated fats [29]. Although it is well documented in models of diabetes and obesity, its expression can also affect lipid composition and influence the production of lipids in the meibomian gland of the eyelid tissues. SREBP-1 regulates fatty acid and triglyceride synthesis [30], and FAS plays a key role in lipogenesis, synthesizing fatty acids from acetyl-CoA and malonyl-CoA [31]. PPAR-γ controls adipogenesis and lipid storage [32]. Animal studies indicate that PPARγ modulation can impact lipid metabolism in tissues subject to metabolic stress [33], which could extend to the regulation of meibomian gland lipid synthesis. In the bioprinted scaffold with hADSC group (group 3), the expression of SREBP-1, FADS-2, and FAS was significantly higher than that in the scaffold-only group, though the PPAR-γ expression did not differ significantly. The hADSCs likely promoted both tissue regeneration and lipid metabolism. hADSCs regulate lipid metabolism via enzymes such as FAS and acetyl-CoA carboxylase, which drive fatty acid synthesis and lipid accumulation when hADSCs differentiate into adipocytes [34]. They also express lipid metabolism genes like SREBP-1 and FADS2, crucial for lipid biosynthesis and desaturation [34]

The aim of this study was to investigate the feasibility of structurally and functionally replacing tarsal plates using a final product that integrates the advantages of 3D-bioprinted scaffolds with hADSCs. The results demonstrated that implanting a scaffold composed of 3D-bioprinted materials embedded with hADSCs can successfully reconstruct the morphology of the eyelids and restore lipid secretion functionality. The characteristics of the tarsal plate significantly contributed to these favorable outcomes. Unlike typical subcutaneous spaces, the tarsal plate is surrounded by dense fibrous tissue rich in blood vessels, which promotes microcirculation [35]. Furthermore, the tarsal plate serves as a reservoir for bioactive signals that can diffuse into the implanted structure, thereby facilitating the transmission of these signals [35]. This characteristic enhances the stability of the transplantation process and mitigates the immune response to the transplanted construct within the tarsal plate microenvironment, ultimately leading to the regeneration of the appropriate type of tissue.

This study has several limitations. First, we did not perform immunohistochemical or Oil Red O staining to determine whether lipid secretion had occurred. Therefore, future studies should focus on lipid secretion through additional experiments. Second, no further experiments were conducted to confirm the tear layer based on lipid secretion or to examine the state of the rat cornea using a slit-lamp microscope. Lastly, the experimental period was relatively short. It would be more valuable to implant 3D-bioprinted scaffolds with MSCs embedded into a rat’s eyelid and observe them under the proper conditions for an extended period. Additionally, a comparative study of the functional features would provide more comprehensive insights.

## 5. Conclusions

This study is significant because it demonstrated the potential of using 3D-bioprinting to replace tarsal plates by embedding hADSCs within scaffolds. An intriguing outcome was the differentiation of the hADSCs into structures resembling lipid-secreting cells within the 3D-printed scaffolds, achieved without the need for additional seeding of sebocytes. The combination of 3D-bioprinted scaffolds and hADSCs proved to be an effective solution for tarsal plate reconstruction in vivo.

## Figures and Tables

**Table 1:** Primer sequences for real-time PCR.

Gene Name	Primer (5′→3′)	Amplicon Size (bp)	GenBank Accession Numbers
* β-actin *	F: CACACCCGCCACCAGTTCG R: ACCCATTCCCACCATCACACC	165	NM 031144.3
*FADS-2*	F: CGTAGCGCACGGGTCATC	296	NM 031344.2
	R: GCTTCAAGAACTTGCCCACG		
*FAS*	F: CCCGGACCCAGAATACCAAG	124	NM 139194.3
	R: GTTCGTGTGCAAGGCTCAAG		
*SREBP-1*	F: GACGACGGAGCCATGGATT	71	NM 001276708
	R: GGGAAGTCACTGTCTTGGTTGTT		
* PPAR-γ *	F: CTGTTCGCCAAGGTGCTCCA	102	XM 063285619.1
	R: GCTCATATCTGTCTCCGTCTTCTT		

*FADS-2*: fatty acid desaturase-2; *FAS*: fatty acid synthase; *SREBP-1*: sterol regulatory element-binding protein-1; *PPAR-γ*: peroxisome proliferator-activated receptor-γ.

**Table 2:** Comparison of the fat-secreting cell counts in all groups ^a^.

	Group 1	Group 2	Group 3	*p*-Value ^b^
Cell counts (cell/10HPF)	177 ± 24.14	26 ± 19.49	137.52 ± 19.56	<0.001

^a^ Values are expressed as means ± SD. ^b^ An ANOVA test was used. *p*-values less than 0.05 were considered statistically significant.

## Data Availability

Data are contained within the article.

## References

[1] Sun M.T., Pham D.T., O’Connor A.J., Wood J., Casson R., Selva D., Costi J.J. (2015). The Biomechanics of eyelid tarsus tissue. J. Biomech..

[2] Liu S., Hatton M.P., Khandelwal P., Sullivan D.A. (2010). Culture, immortalization, and characterization of human meibomian gland epithelial cells. Investig. Ophthalmol. Vis. Sci..

[3] Foulks G.N., Bron A.J. (2003). Meibomian gland dysfunction: A clinical scheme for description, diagnosis, classification, and grading. Ocul. Surf..

[4] Hwang H.S., Parfitt G.J., Brown D.J., Jester J.V. (2017). Meibocyte differentiation and renewal: Insights into novel mechanisms of meibomian gland dysfunction (MGD). Exp. Eye Res..

[5] Zhou J., Peng S.W., Wang Y.Y., Zheng S.B., Wang Y., Chen G.Q. (2010). The use of poly (3-hydroxybutyrate-co-3-hydroxyhexanoate) scaffolds for tarsal repair in eyelid reconstruction in the rat. Biomaterials.

[6] Mannor G.E., Mathers W.D., Wolfley D.E., Martinez J.A. (1994). Hard-palate mucosa graft in Stevens-Johnson syndrome. Am. J. Ophthalmol..

[7] Goldberg R.A., Joshi A.R., McCann J.D., Shorr N. (1999). Management of severe cicatricial entropion using shared mucosal grafts. Arch. Ophthalmol..

[8] Park E., Lewis K., Alghoul M.S. (2017). Comparison of Efficacy and Complications Among Various Spacer Grafts in the Treatment of Lower Eyelid Retraction: A Systematic Review. Aesthet. Surg. J..

[9] Li T.G., Shorr N., Goldberg R.A. (2005). Comparison of the efficacy of hard palate grafts with acellular human dermis grafts in lower eyelid surgery. Plast. Reconstr. Surg..

[10] Scruggs J.T., McGwin Jr G., Morgenstern K.E. (2015). Use of Noncadaveric Human Acellular Dermal Tissue (BellaDerm) in Lower Eyelid Retraction Repair. Ophthalmic Plast. Reconstr. Surg..

[11] Song K., Wang Z., Liu R., Chen G., Liu L. (2018). Microfabrication-Based Three-Dimensional (3-D) Extracellular Matrix Microenvironments for Cancer and Other Diseases. Int. J. Mol. Sci..

[12] Kao C.T., Lin C.C., Chen Y.W., Yeh C.H., Fang H.Y., Shie M.Y. (2015). Poly(dopamine) coating of 3D printed poly(lactic acid) scaffolds for bone tissue engineering. Mater. Sci. Eng..

[13] Yu Y., Moncal K.K., Li J., Peng W., Rivero I., Martin J.A., Ozbolat I.T. (2016). Three-dimensional bioprinting using self-assembling scalable scaffold-free “tissue strands” as a new bioink. Sci. Rep..

[14] Bae S.W., Lee K.W., Park J.H., Lee J., Jung C.R., Yu J., Kim H.Y., Kim D.H. (2018). 3D Bioprinted Artificial Trachea with Epithelial Cells and Chondrogenic-Differentiated Bone Marrow-Derived Mesenchymal Stem Cells. Int. J. Mol. Sci..

[15] Hiller T., Berg J., Elomaa L., Rohrs V., Ullah I., Schaar K., Dietrich A.C., Al-Zeer M.A., Kurtz A., Hocke A.C. (2018). Generation of a 3D Liver Model Comprising Human Extracellular Matrix in an Alginate/Gelatin-Based Bioink by Extrusion Bioprinting for Infection and Transduction Studies. Int. J. Mol. Sci..

[16] Isaacson A., Swioklo S., Connon C.J. (2018). 3D bioprinting of a corneal stroma equivalent. Exp. Eye Res..

[17] Sorkio A., Koch L., Koivusalo L., Deiwick A., Miettinen S., Chichkov B., Skottman H. (2018). Human stem cell based corneal tissue mimicking structures using laser-assisted 3D bioprinting and functional bioinks. Biomaterials.

[18] Chen L., Yan D., Wu N., Zhang W., Yan C., Yao Q., Zouboulis C.C., Sun H., Fu Y. (2020). 3D-Printed Poly-Caprolactone Scaffolds Modified With Biomimetic Extracellular Matrices for Tarsal Plate Tissue Engineering. Front. Bioeng. Biotechnol..

[19] Mutaf M., Temel M. (2017). A New Technique for Total Reconstruction of the Lower Lid. Ann. Plast. Surg..

[20] Atik B., Tan O., Bekerecioglu M., Cinal A., Tekes L. (2007). Reconstruction of lower eyelid defects using a cross upper eyelid flap composited with ear cartilage. Dermatol. Surg..

[21] Matsuzaka Y., Yashiro R. (2022). Therapeutic Strategy of Mesenchymal-Stem-Cell-Derived Extracellular Vesicles as Regenerative Medicine. Int. J. Mol. Sci..

[22] Kim H., Bae C., Kook Y.M., Koh W.G., Lee K., Park M.H. (2019). Mesenchymal stem cell 3D encapsulation technologies for biomimetic microenvironment in tissue regeneration. Stem Cell Res. Ther..

[23] Liu S., Zhou J., Zhang X., Liu Y., Chen J., Hu B., Song J., Zhang Y. (2016). Strategies to Optimize Adult Stem Cell Therapy for Tissue Regeneration. Int. J. Mol. Sci..

[24] Kim H.J., Park J.S. (2017). Usage of Human Mesenchymal Stem Cells in Cell-based Therapy: Advantages and Disadvantages. Dev. Reprod..

[25] Park C.W., Kim K.S., Bae S., Son H.K., Myung P.K., Hong H.J., Kim H. (2009). Cytokine secretion profiling of human mesenchymal stem cells by antibody array. Int. J. Stem Cells.

[26] Cano M.L., Cassimeris L., Joyce M., Zigmond S.H. (1992). Characterization of tetramethylrhodaminyl-phalloidin binding to cellular F-actin. Cell Motil. Cytoskelet..

[27] Gipson I.K., Tisdale A.S. (1997). Visualization of conjunctival goblet cell actin cytoskeleton and mucin content in tissue whole mounts. Exp. Eye Res..

[28] Rice D.S., Hansen G.M., Liu F., Crist M.J., Newhouse M.M., Potter D., Xu N., Abuin A., Vogel P.J., Zambrowicz B.P. (2012). Keratinocyte migration in the developing eyelid requires LIMK2. PLoS ONE.

[29] Jump D.B. (2002). The biochemistry of n-3 polyunsaturated fatty acids. J. Biol. Chem..

[30] Porstmann T., Griffiths B., Chung Y.L., Delpuech O., Griffiths J.R., Downward J., Schulze A. (2005). PKB/Akt induces transcription of enzymes involved in cholesterol and fatty acid biosynthesis via activation of SREBP. Oncogene.

[31] Smith S. (1994). The animal fatty acid synthase: One gene, one polypeptide, seven enzymes. FASEB J..

[32] Tontonoz P., Spiegelman B.M. (2008). Fat and beyond: The diverse biology of PPARgamma. Annu. Rev. Biochem..

[33] Shehnaz S.I., Roy A., Vijayaraghavan R., Sivanesan S. (2023). Luteolin Mitigates Diabetic Dyslipidemia in Rats by Modulating ACAT-2, PPARalpha, SREBP-2 Proteins, and Oxidative Stress. Appl. Biochem. Biotechnol..

[34] Afarin R., Aslani F., Asadizade S., Jaberian Asl B., Mohammadi Gahrooie M., Shakerian E., Ahangarpour A. (2023). The Effect of Lipopolysaccharide-Stimulated Adipose-Derived Mesenchymal Stem Cells on NAFLD Treatment in High-Fat Diet-Fed Rats. Iran. J. Pharm. Res..

[35] Dai Y., Jin K., Feng X., Ye J., Gao C. (2019). Regeneration of different types of tissues depends on the interplay of stem cells-laden constructs and microenvironments in vivo. Mater. Sci. Eng..

